# Feasibility and risks of preoperative embolization for CNS hemangioblastoma surgery at a national VHL referral center

**DOI:** 10.1016/j.bas.2026.107475

**Published:** 2026-10-12

**Authors:** Roberto Doria-Medina, Nicolas Neidert, Marc Hohenhaus, Alexander Rau, Ikram Eda Duman Kavus, Samer Elsheikh, Christian Taschner, Jürgen Beck, Jan-Helge Klingler, Christine Julia Gizaw

**Affiliations:** a Department of Neurosurgery, Medical Center - University of Freiburg, Faculty of Medicine, University of Freiburg, Freiburg, Germany; b Department of Neuroradiology, Medical Center - University of Freiburg, Faculty of Medicine, University of Freiburg, Freiburg, Germany; c Department of Neurosurgery and Spine Surgery, Klinikum Main-Spessart, Lohr Am Main, Germany

**Keywords:** Hemangioblastoma, von Hippel-Lindau disease, Embolization, Feasibility, Complications, Surgical outcomes

## Abstract

**Introduction:**

Preoperative embolization is used selectively for central nervous system hemangioblastomas (CNS HBLs), but anatomical feasibility and procedural risk remain important uncertainties.

**Research question:**

How feasible is embolization in selected CNS HBL cases?

**Material and methods:**

We retrospectively reviewed a prospectively maintained von Hippel-Lindau (VHL)/HBL registry (1997–2025), including operations preceded by angiography with intent to embolize. Feasibility, operator-reported devascularization, complications, and perioperative outcomes were assessed. Devascularization was categorized into “non-feasible”, <50%, 50–75%, and >75%. Comparisons were exploratory.

**Results:**

We identified 237 individuals, 565 operations, and 804 CNS-HBLs. Twenty-seven (4.8%) procedures met inclusion criteria. Embolization was performed in 21/27 cases (77.8%; 95% CI 57.7–91.4%), with >75% devascularization in 13/21 (61.9%). Prohibitive vascular anatomy prevented embolization in six cases. Four angiography-related complications occurred (4/27 overall: 14.8%, 95% CI 4.2–33.7%; 4/21 embolization: 19.0%, 95% CI 5.4–41.9%), including two with permanent neurological deficits. In embolized versus non-feasible cases, median surgery duration was 271 versus 372 min, median blood loss was 425 versus 600 mL, median hemoglobin decrease was 2.6 versus 4.1 g/dL, and functional status improved/remained unchanged/worsened in 10/9/2 versus 0/3/3 cases, respectively. Qualitative bleeding assessments did not differ.

**Conclusions:**

Only a small proportion of CNS HBLs were considered for preoperative embolization. Embolization was feasible in most selected cases but carried relevant procedural risk. The small cohort, and potential confounding prevent attribution of outcome differences to embolization. Careful multidisciplinary selection in high-volume VHL/HBL centers remains essential. Future studies would benefit from standardized angiographic scoring systems and uniform criteria for assessing embolization success.

## Highlights

• Transarterial embolization was feasible in 21 of 27 selected surgical cases.
• Prohibitive vascular anatomy prevented embolization in six cases.
• Four angiography-related complications included two permanent neurological deficits.
• Differences in vascular anatomy limit conclusions about embolization benefit.

## 1 Introduction

Hemangioblastoma (HBL) is a highly vascular tumor of the central nervous system (CNS) that most commonly arises in the cerebellum, brainstem, and spinal cord (Gläsker, 2005). CNS HBLs occur sporadically or in association with von Hippel-Lindau (VHL) disease, an autosomal dominant syndrome caused by pathogenic variants in the VHL tumor-suppressor gene (Latif et al., 1993; Kaelin, 2002). Preoperative endovascular embolization is used selectively with the aim of reducing tumor vascularity before resection. However, indications remain uncertain, and reported risks and outcomes vary widely across small heterogeneous series (Ampie et al., 2016a, 2016b; Palavani et al., 2023; Butenschoen et al., 2023; Cornelius et al., 2007). Differences in tumor location, VHL status, treatment selection, and definitions of devascularization complicate interpretation of the available evidence (Takami et al., 2022; Haruma et al., 2025).

At our national VHL referral center, which provides multidisciplinary care for more than 300 VHL patients annually, we maintain an institutional registry of CNS HBL surgery (Klingler et al., 2020). This study describes operations preceded by diagnostic angiography with intent to embolize, including cases in which embolization was judged unsafe or technically infeasible. We focused on technical feasibility. Functional outcomes are reported descriptively, with exploratory comparisons between groups defined by angiographic feasibility.

## 2 Material and methods

### 2.1 Study design and data collection

We performed a retrospective analysis of our prospectively maintained VHL/HBL database (Klingler et al., 2020), managed in REDCap (Research Electronic Data Capture). The registry includes patients who underwent surgery for at least one CNS HBL at our center. Clinical and imaging data were entered by the VHL team during routine outpatient surveillance visits and hospital admissions. The patient outcomes and complication follow-up were similarly assessed from the dedicated VHL team. For this study, we screened all records from January 1, 1997, to July 31, 2025.

We included all CNS HBL operations preceded by transarterial diagnostic cerebral or spinal angiography with intent to perform preoperative tumor embolization, regardless of whether embolization was ultimately achieved. Feasibility was abstracted from the interventional reports and classified as embolized or non-feasible. Devascularization was the treating operator's angiographic estimate of the reduction in tumor vascularity, as documented in the interventional report. These report-based estimates were grouped retrospectively as: (1) <50% devascularization (low), (2) 50–75% (partial), and (3) >75% (near-complete). Comparisons across all four treatment categories also included (4) non-feasible cases, in which no embolic agent was administered.

Baseline and perioperative variables included age, sex, VHL status, operative duration, interval from angiography with intent to embolize to surgery, estimated blood loss (EBL), and red blood cell (RBC) transfusion. Hemoglobin (Hb) was obtained immediately before surgery and from the first postoperative measurement (median 1 day, range 0–1 days). The perioperative decrease was calculated as ΔHb. Functional status was recorded before surgery using the modified Rankin Scale (mRS). For survivors, postoperative mRS was assessed at the follow-up associated with the postoperative MRI. Postoperative death was coded as mRS 6. Improvement, no change, and worsening were defined by a decrease, no change, or increase in the recorded mRS, respectively. Tumor characteristics included location, compartment, and volume, estimated as A×B×C/2, where A, B, and C represented the maximum orthogonal tumor diameters. Recorded volumes in mm^3^ were converted to mL. Extent of resection was obtained from the postoperative MRI report.

Operative reports were reviewed for descriptions of the surgical field. Assessments were classified as favorable (for example, reduced bleeding or improved dissection planes) or unfavorable (for example, persistent hypervascularity or excessive bleeding). This qualitative endpoint reflects the operative-report description and is distinct from measured EBL. These retrospective assessments were related to embolization status and devascularization category.

For this analysis, angiography-related complications comprised documented vascular-access injury, catheter-related or nontarget arterial injury, and tumor hemorrhage occurring during or immediately after angiography with intent to embolize. Events were counted irrespective of whether they caused lasting sequelae. Hemorrhage arising during or after subsequent surgery in a non-feasible case was described as surgical adverse event and was not included in the angiography-related complication count.

The temporal distribution of cases was summarized by operation year within the periods of 1997–1999, 2000–2009, 2010–2019, and 2020–2025. Individual case characteristics, documented embolic agents, devascularization categories, complications, and preoperative and postoperative mRS are provided in Supplementary Table S2.

### 2.2 Surgical planning and embolization technique

Surgical indications were routinely discussed at an interdisciplinary tumor conference. When preoperative embolization was considered, the case was reviewed with interventional neuroradiologists before angiography. The decision to proceed with transarterial embolization depended on the vascular anatomy demonstrated during the procedure.

Among the 21 embolized cases, 19 (90.5%) were treated with particles and two (9.5%) with Onyx. The most frequently used particles were Contour 150–250 μm (7/19), Contour 250–355 μm (5/19), and Contour 45–150 μm (3/19). Four particles were not specified.

### 2.3 Statistical analysis

The analysis emphasizes descriptive reporting of transarterial feasibility and procedural complications. Continuous variables are summarized as medians with interquartile ranges (IQR) or ranges, as indicated. Categorical variables are reported as counts and percentages. Between-group comparisons of continuous variables used the Mann–Whitney *U* test, and categorical comparisons used Fisher's exact test. Paired preoperative and postoperative values were compared using the Wilcoxon signed-rank test. The distribution of improved, unchanged, and worsened mRS was compared using a 2×3 Fisher–Freeman–Halton exact test. All tests were two-sided. Reported p values are exploratory. The groups were defined by angiographic feasibility, and these comparisons cannot estimate a causal treatment effect. Exact two-sided 95% confidence intervals for feasibility and complication proportions were calculated using the Clopper–Pearson method. A post hoc influence analysis repeated the three-category functional comparison after excluding the single postoperative death. The death remained included in the main analysis. Analyses used available observations for each outcome without imputation.

Statistical analyses were performed in R (Version 4.6.1) and RStudio (Posit PBC, Version, 2026.08.0 + 187) using the packages tidyverse, scales, gridExtra, and cowplot. Exact binomial confidence intervals and the post hoc influence analysis were calculated using Python 3.12.14, with fixed-margin enumeration for the Fisher–Freeman–Halton test.

## 3 Results

### 3.1 Embolization feasibility

The registry included 237 individuals who underwent 565 operations for 804 CNS-HBLs. Twenty-seven operations in 27 patients were preceded by angiography with intent to embolize. Embolization was performed in 21/27 cases (77.8%; 95% CI 57.7–91.4%). In six (22.2%), angiography identified anatomy that precluded embolization. Among embolized cases, devascularization was >75% in 13/21 (61.9%), 50–75% in 6/21 (28.6%), and <50% in 2/21 (9.5%). The cohort comprised 16 spinal cases (13 embolized and three non-feasible) and 11 intracranial cases (eight embolized and three non-feasible). Median tumor volume was 2.2 mL (IQR 1.7–2.9) for spinal lesions and 7.6 mL (IQR 5.6–17.1) for intracranial lesions. Greater than 75% devascularization was recorded in 10/16 spinal and 3/11 intracranial cases (Table 1). Surgery was performed on the day of the angiography in 2/27 cases, one day later in 20/27, and two days later in 5/27 (median interval 1 day, range 0–2 days).

**Table 1:** Characteristics of the included surgical episodes.

(a) By feasibility	Embolization n = 21	Non-feasible n = 6	P value
Age, y, median (range)	43 (21–74)	45 (21–65)	0.85
Sex, male/female, n (%)	11 (52.4)/10 (47.6)	5 (83.3)/1 (16.7)	0.35
VHL, n (%)	14 (66.7)	5 (83.3)	0.63
Tumor volume, mL, median (IQR)	3.4 (2.2–7.6)	2.0 (1.4–4.7)	0.19
Tumor morphology	N (%)	N (%)	0.32
Solid tumor	15 (71.4)	3 (50)	
Extratumoral cyst	4 (19)	1 (16.7)	
Intratumoral cyst	2 (9.5)	2 (33.3)	
Devascularization rate			
Near-complete	13 (61.9)		
Partial	6 (28.6)		
Low	2 (9.5)		

(b) By tumor location	Spinal n = 16	Intracranial n = 11	P value
Tumor volume, mL, median (IQR)	2.2 (1.7–2.9)	7.6 (5.6–17.1)	0.001
Devascularization rate	N (%)	N (%)	0.27
Near-complete	10 (62.5)	3 (27.3)	
Partial	2 (12.5)	4 (36.4)	
Low	1 (6.3)	1 (9.1)	
Non-feasible	3 (18.8)	3 (27.3)	

(a) Characteristics of cases in which embolization was performed or was judged unsafe or infeasible. (b) Distribution of devascularization categories by tumor location. Percentages use surgical episodes as denominators. Devascularization was operator-reported: low (<50%), partial (50–75%), or near-complete (>75%). The location-specific p value for devascularization compares all four categories. All statistical comparisons are exploratory and non-significant results do not establish group comparability.

Abbreviations: HBL, hemangioblastoma; VHL, von Hippel-Lindau; IQR, interquartile range.

The 27 included operations took place between 2005 and 2024. No cases occurred in 1997–1999; two occurred in 2000–2009, 12 in 2010–2019 and 13 in 2020–2025. The corresponding numbers of embolized/non-feasible cases were 0/0, 2/0, 11/1, and 8/5. Twenty-five operations (92.6%) occurred from 2010 onward. Supplementary Table S2 provides the individual case listings.

### 3.2 Anatomical reasons for withholding embolization

In six cases, angiography demonstrated anatomy that made embolization unsafe or technically infeasible. Constraints included multiple small feeders, shared supply to the spinal cord or brainstem, hazardous anastomoses, and inability to obtain a catheter position that would avoid reflux into eloquent vessels. Three cases were spinal and three intracranial, including two lesions at the obex. In one spinal case, a second angiographic attempt on the following day was also unsuccessful (Table 2a).

**Table 2:** Anatomical constraints and angiography-related complications.

(a) Cases in which embolization was judged unsafe or infeasible			
*Case*	*Tumor location*	*Devascularization rate*	*Description*

1	Infratentorial, intramedullary at obex	Non-feasible	Multiple small pathological feeders from the left vertebral artery and right PICA. No safe transarterial embolization route.
2	Intramedullary at C5	Non-feasible	Tumor feeders supplied the spinal cord. Right vertebral artery (proximal V1) with anastomosis to a radiculomedullary artery to the anterior spinal artery.
3	Intracranial, left cerebellar paramedian	Non-feasible	Multiple feeders. Proximity to brainstem and left internal acoustic meatus prevented a catheter position that would avoid reflux into eloquent vessels.
4	Intramedullary at C5–C7	Non-feasible	Predominant supply from right costocervical trunk, with dominant contribution from the cervicothoracic anterior spinal artery within the tumor region.
5	Intramedullary at T8	Non-feasible	Supply via a radiculomedullary artery from left T10. Second attempt on the following day also not feasible.
6	Infratentorial, extramedullary at obex	Non-feasible	Multiple passing vessels supplying cerebellar parenchyma. Particle embolization unsafe.

(b) Qualitative analysis of angiography-related complications			
*Case*	*Tumor location*	*Devascularization rate*	*Description*

7	Extramedullary at C6/C7	Near-complete	Dissection of the femoral artery. No sequelae.
8	Intracranial, right cerebellar lateral	Partial	Labyrinthine artery injury with hypoacusis, facial asymmetry, and hemifacial hypesthesia. Permanent neurological deficit, but manageable; mRS increased from 2 to 3.
9	Intra-/extradural, extramedullary, right L3	Low	Perforation of a small lumbar arterial branch with contrast extravasation. No sequelae.
10	Intracranial, medulla oblongata to obex	Partial	Acute tumoral hemorrhage with intraventricular breach. Urgent surgical decompression required. Permanent neurological deficit; mRS increased from 3 to 5.

(a) Embolization was withheld because of shared supply to eloquent cord or brainstem territories, hazardous anastomoses, or inability to obtain a catheter position that would prevent reflux. (b) Events occurred during or immediately after angiography with embolization. Cases 7 and 9 had vascular injuries without reported sequelae. Cases 8 and 10 had permanent neurological deficits. Devascularization was operator-reported: low (<50%), partial (50–75%), or near-complete (>75%).

Abbreviations: PICA, posterior inferior cerebellar artery; V1, the prevertebral (V1) segment of the vertebral artery.

### 3.3 Procedure-related complications

Four angiography-related complications occurred (14.8%; 95% CI 4.2–33.7%). All four occurred among the 21 embolized cases (19.0%; 95% CI 5.4–41.9%). Two vascular injuries, femoral artery dissection and lumbar arterial branch perforation, occurred in the spinal cases and caused no reported sequelae (cases 7 and 9 in Table 2b). Two events caused permanent neurological deficits: labyrinthine artery injury with hearing and facial deficits, and intraprocedural tumor hemorrhage with intraventricular extension requiring urgent surgical decompression (cases 8 and 10 in Table 2b). Both occurred in intracranial cases, corresponding to 2/8 intracranial embolizations. Permanent neurological deficits occurred in 2/27 cases overall (7.4%; 95 CI 0.9–24.3%) and 2/21 embolized cases (9.5%; 95% CI 1.2–30.4%). No procedure-related neurological deficit was recorded among the 13 embolized spinal cases.

### 3.4 Exploratory surgical outcomes

Median operative duration was 271 min (IQR 230–332) in embolized cases and 372 min (IQR 345–439) in non-feasible cases (P = 0.031). EBL was available for 18/21 embolized and 6/6 non-feasible cases. Median EBL was 425 mL (IQR 263–1125) and 600 mL (IQR 340–1100), respectively (P = 0.79). Complete resection was recorded in 17/20 evaluable embolized cases (85%) and 6/6 non-feasible cases (100%; P = 1.00). Extent-of-resection assessment was unavailable for one embolized case (Supplementary Table S1). RBC transfusion was recorded in 0/21 embolized cases and in 1/6 non-feasible cases; the latter received seven units.

Median preoperative Hb was 14.6 versus 14.7 g/dL (P = 0.84), and postoperative Hb was 11.4 versus 10.9 g/dL (P = 0.88), in embolized and non-feasible cases, respectively. Median ΔHb was 2.6 g/dL (IQR 1.9–4.3) versus 4.1 g/dL (IQR 3.2–4.6; P = 0.30). One postoperative Hb value was missing in the embolized group; paired Hb measurements and ΔHb were available for 20/21 embolized and 6/6 non-feasible cases (Fig. 1).

**Fig. 1:**
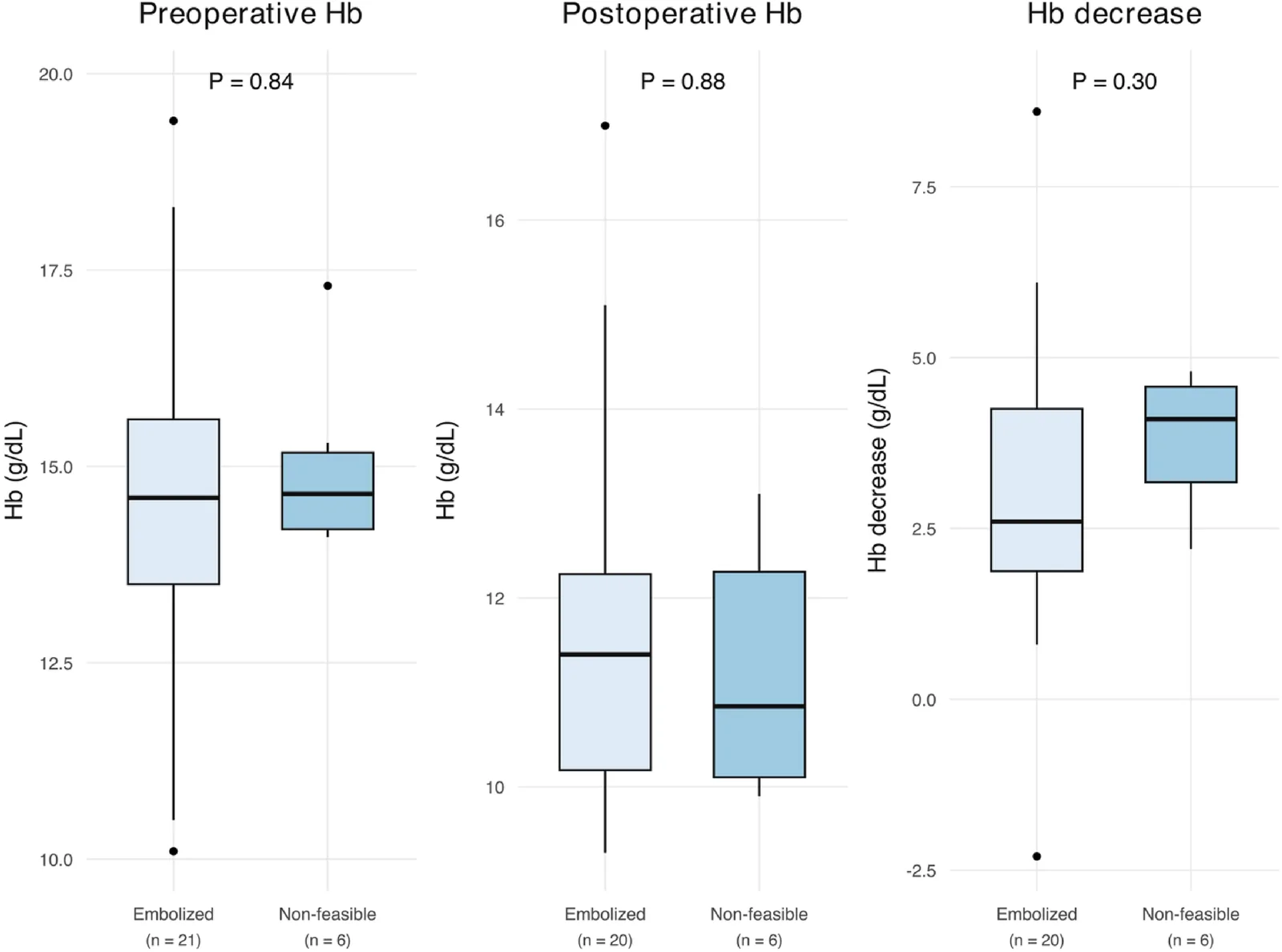
Perioperative hemoglobin by embolization status. Preoperative and postoperative Hb by embolization status. Boxes show medians and IQRs. Median ΔHb was 2.6 g/dL (IQR 1.9–4.3; n = 20) in embolized cases and 4.1 g/dL (IQR 3.2–4.6; n = 6) in cases not embolized because of non-feasibility. The between-group comparison of ΔHb yielded P = 0.30. One postoperative Hb value was missing in the embolized group and was excluded from paired analyses. All statistical comparisons are exploratory. Abbreviations: Hb, hemoglobin.

The operative field was described as favorable in 17/27 cases (63.0%) and unfavorable in 10/27 cases (37.0%). Exploratory comparisons yielded P = 1.00 across the four devascularization categories and P = 1.00 for embolized versus non-feasible cases (Supplementary Fig. S1). These retrospective assessments did not demonstrate a relationship between devascularization grade and the recorded operative-field assessment.

### 3.5 Exploratory functional outcomes

Among 21 embolized cases, postoperative mRS improved relative to the preoperative assessment in 10 (47.6%), remained unchanged in nine (42.9%), and worsened in two (9.5%). Among six non-feasible cases, none improved, three (50.0%) were unchanged, and three (50.0%) worsened. The exploratory comparison of the full improved/unchanged/worsened distribution yielded P = 0.031 (2 × 3 Fisher–Freeman–Halton exact test). This P value refers to the three-category distribution, not to improvement alone. Within-group paired comparisons yielded P = 0.078 for embolized cases and P = 0.174 for non-feasible cases (Wilcoxon signed-rank test). These results describe outcomes in anatomically selected groups and cannot establish an effect of embolization (Fig. 2.). In the post hoc influence analysis excluding the postoperative death, the remaining five non-feasible cases included three with unchanged mRS and two with worsened mRS; none improved. The three-category comparison yielded P = 0.074. This analysis assesses the influence of one event.

**Fig. 2:**
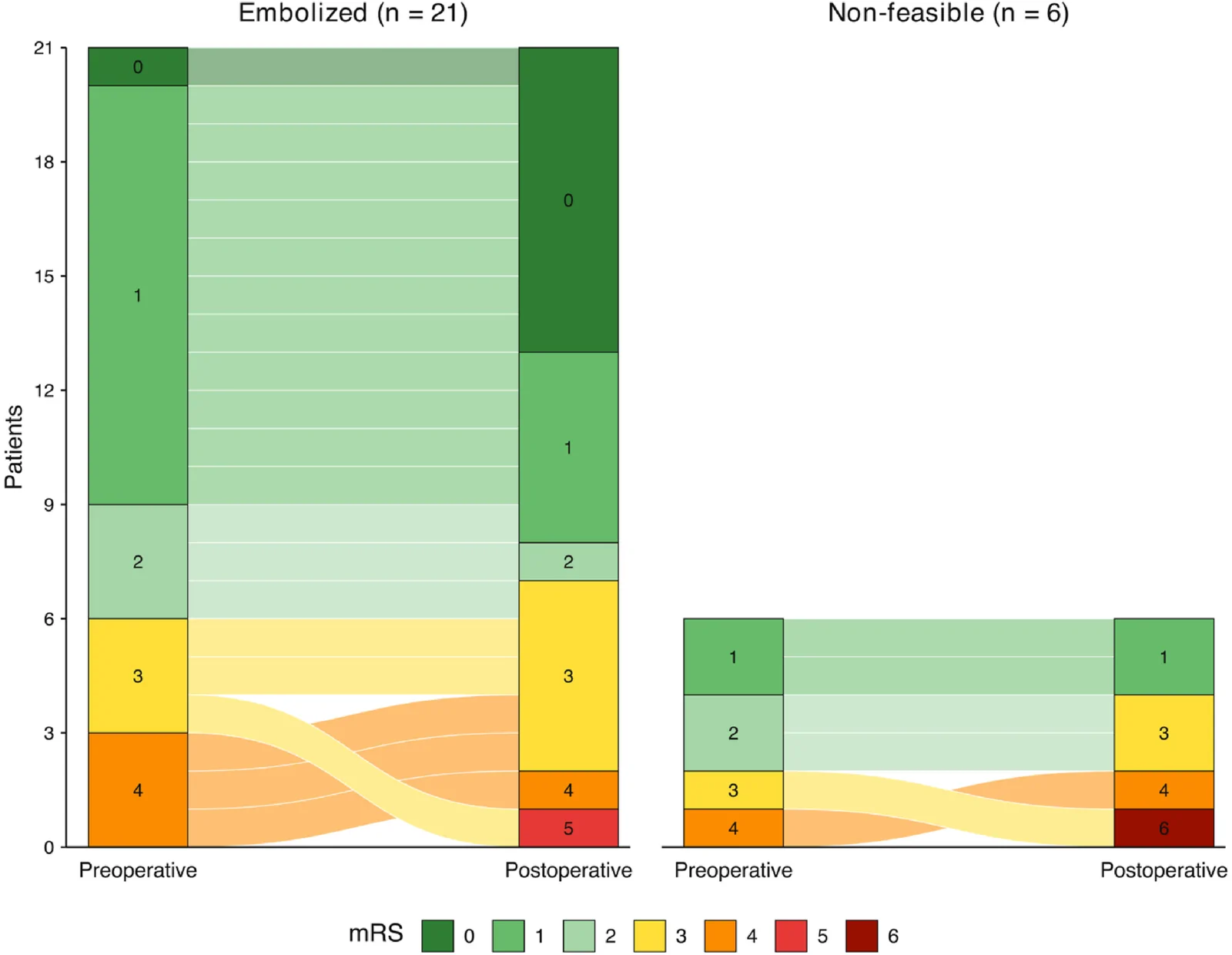
Preoperative and postoperative functional grade by embolization status. Preoperative-to-postoperative mRS transitions for surgical episodes with embolization (left) and episodes in which embolization was judged infeasible (right). Colors indicate mRS categories. In the embolized group, 10/21 cases improved, 9/21 were unchanged, and 2/21 worsened. In the non-feasible group, 0/6 improved, 3/6 were unchanged, and 3/6 worsened. The exploratory comparison of this three-category distribution yielded P = 0.031 (2 × 3 Fisher–Freeman–Halton exact test). Within-group paired comparisons yielded P = 0.078 and P = 0.174, respectively (Wilcoxon signed-rank test). Abbreviations: mRS, modified Rankin Scale.

### 3.6 Illustrative adverse events

In one non-feasible case (case 6 in Table 2; Fig. 3A–D), massive intraoperative hemorrhage occurred (EBL 2740 mL) requiring seven units of packed RBCs and administration of fresh frozen plasma, prothrombin complex concentrate, and fibrinogen. Despite hemostasis after tumor resection, acute postoperative hemorrhage caused brainstem compression and intraventricular extension. Emergency hematoma evacuation and external ventricular drainage were performed. MRI on the following day demonstrated irreversible brainstem injury. After transition to supportive care, the patient died several days later. This death is included in the reported functional outcomes.

**Fig. 3:**
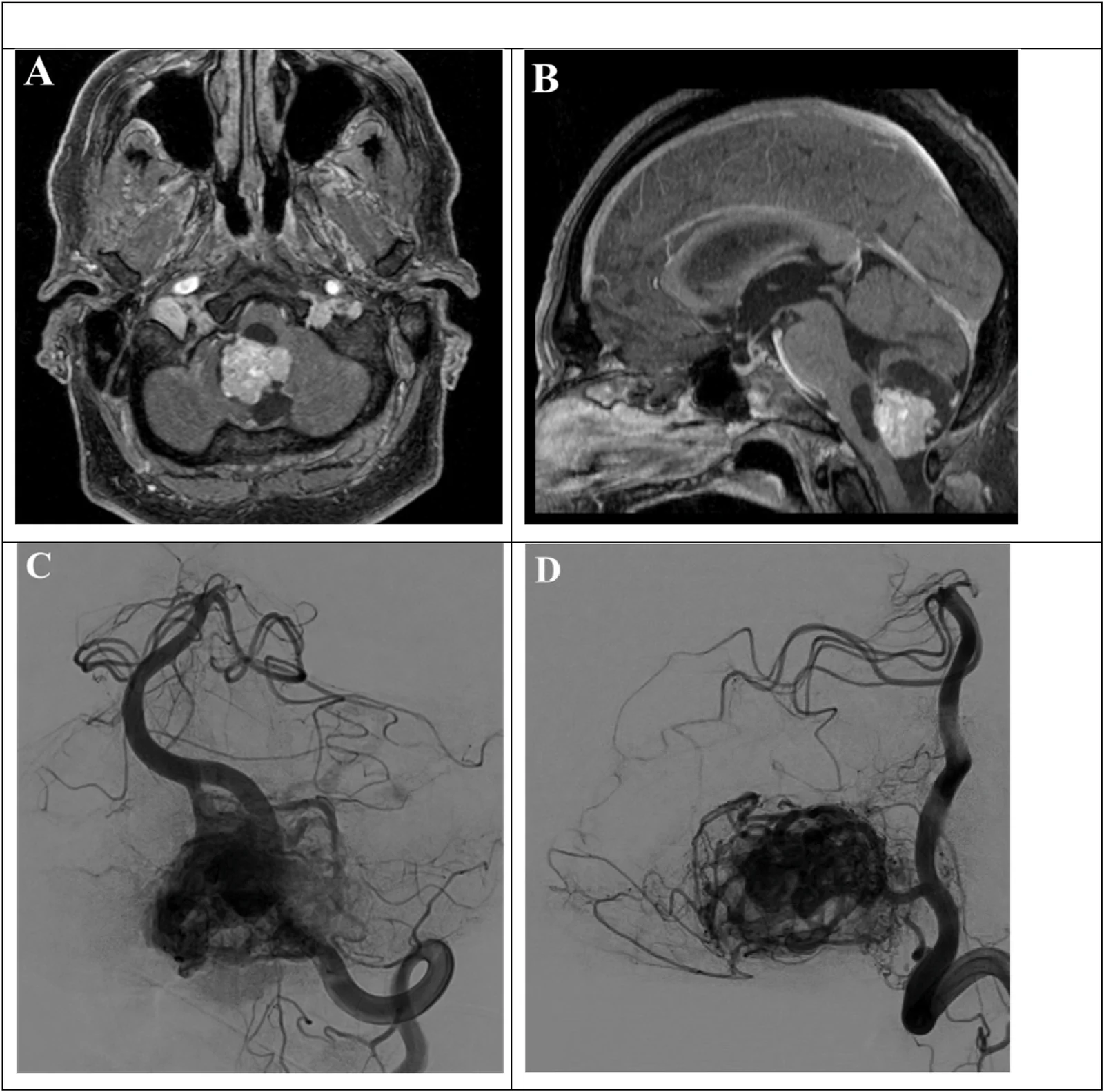

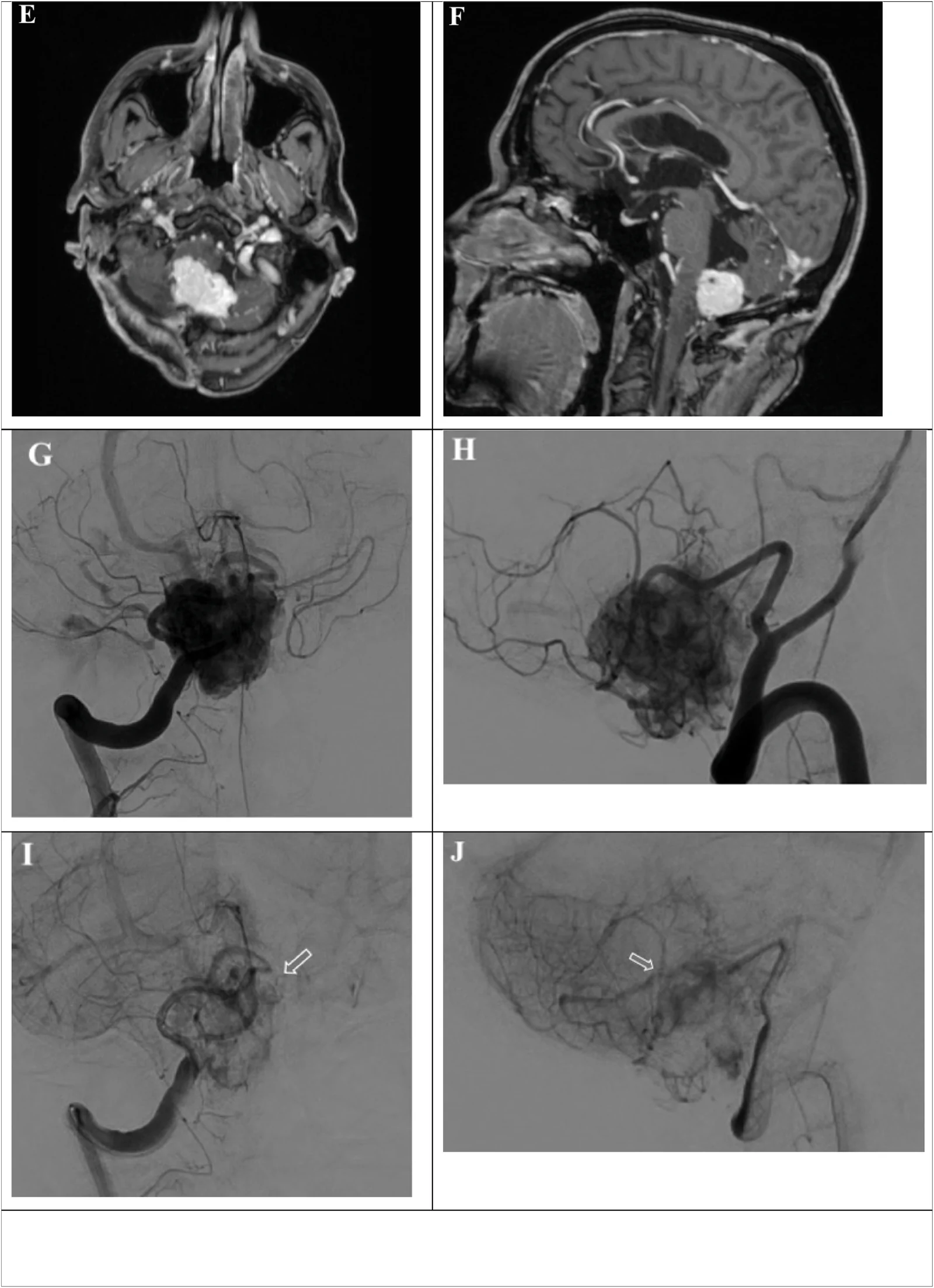
A–J Perioperative imaging of two cases with adverse events. (A–D) Case 6: axial and sagittal post-contrast T1-weighted MRI show a hemangioblastoma at the obex (A, B). Angiography demonstrated multiple en-passant feeders that precluded safe embolization (C, D). In this case, particle embolization was deemed non-feasible due to multiple passing vessels supplying cerebellar parenchyma. The patient suffered massive intraoperative hemorrhage. Despite hemostasis after tumor resection, acute postoperative hemorrhage caused brainstem compression and intraventricular extension. Emergency hematoma evacuation and external ventricular drainage were performed. MRI on the following day demonstrated irreversible brainstem injury. After transition to supportive care, the patient died several days later. This death is included in the reported functional outcomes but was not counted as a angiographic-related complication. (E–J) Case 10: axial and sagittal post-contrast high-resolution T1-weighted MRI show a hemangioblastoma at the obex (E, F). Angiography before (G, H) and after (I, J) embolization demonstrates substantial devascularization with residual superior tumor blush (arrows). In this case, partial particle embolization with Contour 45–150 μm was achieved. However, the patient suffered a post-interventional acute tumoral hemorrhage with intraventricular breach which required urgent surgical decompression. The patient deteriorated from a preoperative mRS of 3 to mRS 5.

In another case (case 10 in Table 2; Fig. 3E–J), partial embolization was complicated by intraprocedural tumor hemorrhage, recognized after delayed emergence from anesthesia. The patient underwent urgent decompression and tumor resection, preceded by external ventricular drain placement. Hemostasis was secured with a clip. EBL was 1300 mL; no RBC transfusion was required. Complete tumor resection was achieved, and postoperative mRS worsened from 3 to 5.

## 4 Discussion

This series describes transarterial embolization feasibility, anatomical constraints, procedural complications, and postoperative outcomes in 27 selected CNS HBL operations. Embolization was performed in 21 cases, while angiography identified anatomy that precluded safe transarterial treatment in six. The latter cases constitute a subgroup selected by vascular anatomy and cannot serve as a matched control cohort. Because the anatomy determining feasibility may also influence surgical difficulty and outcome, between-group differences cannot establish a treatment effect.

The surgical challenge reflects the vascularity of HBL and the relationship between tumor feeders, draining veins, and adjacent neural structures. As with other hypervascular lesions including arteriovenous malformations and angiomas, microsurgical descriptions emphasize control of arterial supply while preserving venous drainage until late in resection (Samartzis et al., 2016; Wan et al., 2011). Embolization may be considered when vascular control is expected to be difficult, but its own risks and the anatomical selection of treated cases complicate assessment of clinical benefits (Ampie et al., 2016a, 2016b; Cornelius et al., 2007).

### 4.1 Feasibility and alternative access routes

Embolization was achieved in 21/27 cases (77.8%) with >75% devascularization in 13/21 embolized cases (61.9%). Published series describe substantial variations in devascularization and treatment selection (Palavani et al., 2023; Wu et al., 2013; Li et al., 2021; Takeuchi et al., 2001; Sultan et al., 2016). Takeuchi et al. reported cases with incomplete or no embolization in which intended embolization could not be performed because safe feeder catheterization was not possible and achieved >75% devascularization in 3/8 patients (Takeuchi et al., 2001). The multicenter spinal HBL series by Butenschoen et al. included six embolized cases among 18 undergoing angiography (Butenschoen et al., 2023). Sultan et al. reported total devascularization in 6/10 intracranial HBLs (Sultan et al., 2016). Li et al. reported a mean of 62% in 7 patients with CNS HBLs with two cases achieving 80–85%, and two cases achieving 70% devascularization rate (Li et al., 2021). Wu et al. described devascularization rates of 40–100% in all 11 included patients with 4 achieving 60–100% and 7 achieving 40–60% (Wu et al., 2013). Haruma et al. reported total/subtotal/partial/unchanged rates of 13.5%/45.4%/39.7%/0.7% for intracranial HBL, a lower total devascularization rate compared to meningiomas (Haruma et al., 2025). Differences in anatomy, referral criteria, and definitions of technical success limit comparison of these proportions. Reporting all cases considered for embolization, including the reasons for withholding treatment, is therefore essential.

Non-feasibility in our series refers specifically to the transarterial approach. Scarcia et al. described balloon-protected direct-puncture embolization of a medulla oblongata HBL using Onyx 18 (Scarcia et al., 2024). This single-case technical report describes an alternative access strategy but does not establish its general safety or comparative effectiveness. Whether the six non-feasible cases in our cohort, including the two obex lesions, would have been suitable for direct puncture cannot be determined.

### 4.2 Interval to surgery

Surgery followed angiography with intent to embolize after a median of one day, reflecting institutional practice. This interval describes all 27 included cases, including those non-feasible cases. The narrow timing range and small cohort do not permit conclusions about the optimal interval between embolization and CNS HBL resection.

Surgical timing after embolization has been discussed extensively, although mainly in other contexts (Kato et al., 2013; Yuh et al., 2023). In studies of other hypervascular spinal tumors, most authors recommend early surgery after embolization. Kato et al. (2013) reported higher EBL when surgery was performed later rather than earlier. It was discussed whether this was due to rapid revascularization. However, this effect may be less relevant when only partial embolization is achieved, although the impact on EBL is then also expected to be smaller (Yuh et al., 2023). In a systematic review and meta-analysis of 1469 cases of preoperative embolization for arteriovenous malformations, shorter intervals of < 48 h were associated with fewer postembolization complications and more complete resections (Gülsuna et al., 2026). The authors concluded that delay should be minimized. A proposed rationale for early surgery is that postponement may allow the development of perilesional edema and partial recanalization of arterial feeders (Winkler et al., 2018, 2022).

### 4.3 Surgical and functional outcomes

Operative duration was shorter in embolized cases, whereas the analyses did not detect between-group differences in EBL or perioperative Hb change. These exploratory comparisons remain vulnerable to differences in vascular anatomy, tumor characteristics, location, baseline function, and treatment era. Non-significant findings do not demonstrate equivalence, and the shorter observed operative duration cannot be attributed to embolization.

Published HBL reports and reviews similarly include heterogeneous, selected populations and differing approaches to outcome assessment (Ampie et al., 2016a; Palavani et al., 2023; Li et al., 2021; Sultan et al., 2016; Sakamoto et al., 2012; Ene et al., 2016). Estimates from such cohorts require careful interpretation because factors determining embolization may determine operative risk. Benefits of preoperative embolization have been reported across other selected hypervascular spinal tumors (Griessenauer et al., 2016; Al-Mistarehi et al., 2025); its impact in CNS HBL might differ compared to other tumors because of its distinct vascular architecture and biological behaviors (Ampie et al., 2016a, 2016b; Haruma et al., 2025).

The distribution of mRS change differed between the feasibility groups in an exploratory analysis, but this cannot establish a functional benefit of embolization. The six-case non-feasible group included one postoperative death and two other cases with worsening mRS. A single severe event has substantial influence in a group of this size, and the death remains part of the principal outcome description. The post hoc comparison excluding the death yielded P = 0.074, illustrating the sensitivity of inference to a single event. Two embolized cases worsened after procedure-related neurological complications. The non-significant within-group paired tests address a different question from the between-group comparison of change categories. Neither resolves confounding by anatomy.

### 4.4 Procedure-related complications

Angiography-related complications occurred in 4/27 included cases (14.8%) and 4/21 embolized cases (19.0%). The femoral artery dissection and lumbar arterial branch perforation caused no reported sequelae. The labyrinthine artery injury and tumor hemorrhage caused permanent neurological deficits. Both permanent deficits occurred among the eight embolized intracranial cases. No procedure-related neurological deficits was recorded among the 13 embolized spinal cases. Published HBL studies report heterogeneous complication rates and definitions (Ampie et al., 2016a, 2016b; Palavani et al., 2023; Cornelius et al., 2007; Ene et al., 2016). In the Japanese neuroendovascular registry, Haruma et al. reported complications in 21/141 (14.9%) intracranial HBL embolizations (Haruma et al., 2025). Differences in anatomy, technique, and event ascertainment limit direct comparison. With only four events, our series cannot reliably identify predictors of complications.

Compared with other intracranial tumor entities, the complication risk in HBL appears higher. According to Haruma et al., the overall complication rate across 2603 intracranial tumors was 4.8%, whereas HBL showed a rate of approximately 3.5 times that of meningioma (4.3%) (Haruma et al., 2025). In two systematic reviews by Ampie et al., embolization of intracranial HBLs appeared to carry a higher complication risk than embolization of spinal HBLs (Ampie et al., 2016a, 2016b).

### 4.5 Clinical implications

The six non-feasible cases document anatomical constraints encountered during angiography, including shared supply to eloquent tissue and catheter positions associated with unacceptable reflux risk. These observations support documenting the reasons for withholding transarterial treatment alongside the achieved degree of devascularization. Notably, in one patient for whom embolization was non-feasible, massive perioperative hemorrhage with irreversible brainstem injury and death despite rescue surgery occurred (Table 2 and Fig. 3A–D). This creates a clinical catch-22: more complex tumors may perhaps benefit most from embolization, yet they carry higher procedural risk. However, the fatal hemorrhage after this non-feasible case illustrates the severity of surgical risk in selected lesions but does not establish that embolization would have prevented the event.

Prospective studies should record the intended approach, vascular anatomy, reasons for non-treatment, embolic agents, procedure-related events, and standardized functional outcomes. Assessment of clinical effectiveness will require comparable treatment groups to address confounding.

### 4.6 Strengths and limitations

Strengths include a prospectively maintained institutional registry, multidisciplinary VHL/HBL care, and inclusion of cases in which intended embolization was not performed. The detailed non-feasible cases provide information that would be absent from a series restricted to completed embolizations.

Limitations include the retrospective analysis, a small and highly selected cohort, and anatomical selection that prevents a causal comparison between embolized and non-feasible cases. Numerous exploratory comparisons increase the risk of chance findings, and neither non-significant baseline comparisons nor a shared initial rationale establishes exchangeability of the groups. Registry screening covered 1997–2025, but the included operations occurred during 2005–2024, with 25/27 performed from 2010 onward. The series also predominantly reflects particle embolization. Changes in patient selection, patient equipment, embolic materials, and perioperative care may have influenced feasibility and outcomes. All six non-feasible cases occurred from 2019 onwards, so feasibility groups also differed in their temporal distribution. Devascularization was operator-reported and operative-field assessments were qualitative. Because inclusion required a subsequent operation, these data describe the included surgical cohort rather than every possible event after referral for angiography.

## 5 Conclusions

In this selected single-center cohort within a multidisciplinary national VHL referral center, transarterial embolization was performed in 21 of 27 CNS HBL operations. Anatomical constraints prevented embolization in six cases. Four angiography-related complications occurred, all among embolized cases, including two vascular injuries without reported sequelae and two events causing permanent neurological deficits. The study describes technical feasibility, reasons for withholding embolization, and procedure-related harm. Differences in surgical and functional outcomes between anatomically selected groups cannot establish a benefit of embolization. Standardized anatomical descriptions, transparent patient and procedural denominators, and prospective outcome assessment are needed to improve the evidence base.

## Author contributions

All authors contributed to the study conception and design. Material preparation, data collection and analysis were performed by Roberto Doria-Medina, Christine Julia Gizaw, Jan-Helge Klingler, Marc Hohenhaus, and Nicolas Neidert. The first draft of the manuscript was written by Roberto Doria-Medina and Christine Julia Gizaw, and all authors commented on previous versions of the manuscript. All authors read and approved the final manuscript.

## Ethics approval

This study was performed in line with the principles of the Declaration of Helsinki. Approval was granted by the Ethics Committee of the University of Freiburg (No 21-1277). Informed consent was obtained from all individual participants included in the study.

## Clinical trial number

Not applicable.

## Declaration of generative AI and AI-assisted technologies in the manuscript preparation process

During preparation of this work, the authors used AI tools to improve language and readability. After using this service, the authors reviewed and edited the content as needed and take full responsibility for the content of the published article.

## Funding

We acknowledge support by the Open Access Publication Fund of the 10.13039/501100002714University of Freiburg.

## Declaration of competing interest

The authors declare that they have no known competing financial interests or personal relationships that could have appeared to influence the work reported in this paper.

## References

[bib1] Al-Mistarehi A.-H., Slika H., El Baba B. (2025). Optimizing surgical strategies: a systematic review of the effectiveness of preoperative arterial embolization for hyper vascular metastatic spinal tumors. Spine J..

[bib3] Ampie L., Choy W., Khanna R. (2016). Role of preoperative embolization for intradural spinal hemangioblastomas. J. Clin. Neurosci..

[bib2] Ampie L., Choy W., Lamano J.B. (2016). Safety and outcomes of preoperative embolization of intracranial hemangioblastomas: a systematic review. Clin. Neurol. Neurosurg..

[bib4] Butenschoen V.M., Schwendner M., Hubertus V. (2023). Preoperative angiographic considerations and neurological outcome after surgical treatment of intradural spinal hemangioblastoma: a multicenter retrospective case series. J. Neuro Oncol..

[bib5] Cornelius J.F., Saint-Maurice J.P., Bresson D. (2007). Hemorrhage after particle embolization of hemangioblastomas: comparison of outcomes in spinal and cerebellar lesions. J. Neurosurg..

[bib6] Ene C.I., Xu D., Morton R.P. (2016). Safety and efficacy of preoperative embolization of intracranial hemangioblastomas. Oper. Neurosurg..

[bib7] Gläsker S. (2005). Central nervous system manifestations in VHL: genetics, pathology and clinical phenotypic features. Fam. Cancer.

[bib8] Griessenauer C.J., Salem M., Hendrix P. (2016). Preoperative embolization of spinal tumors: a systematic review and meta-analysis. World Neurosurg..

[bib9] Gülsuna B., Gülsuyu B., Aksoy T. (2026). Optimal timing of microsurgical resection after preoperative embolization of intracranial arteriovenous malformations: a systematic review and meta-analysis of 1,469 patients. Neurosurg. Rev..

[bib10] Haruma J., Sugiu K., Hishikawa T. (2025). Real-world experience of embolization for intracranial tumors in Japan: analysis of 2,756 cases from Japanese registry of NeuroEndovascular therapy 4. Neurol. Med.-Chir..

[bib11] Kaelin W.G. (2002). Molecular basis of the VHL hereditary cancer syndrome. Nat. Rev. Cancer.

[bib12] Kato S., Hozumi T., Takaki Y. (2013). Optimal schedule of preoperative embolization for spinal metastasis surgery. Spine.

[bib13] Klingler J.-H., Gläsker S., Bausch B. (2020). Hemangioblastoma and von Hippel-Lindau disease: genetic background, spectrum of disease, and neurosurgical treatment. Childs Nerv. Syst..

[bib14] Latif F., Tory K., Gnarra J. (1993). Identification of the von Hippel-Lindau disease tumor suppressor gene. Science.

[bib15] Li P., Tian Y., Song J. (2021). Microsurgical intracranial hypervascular tumor resection immediately after endovascular embolization in a hybrid operative suite: a single-center experience. J. Clin. Neurosci..

[bib16] Palavani L.B., Andreão F.F., de Abreu L.V. (2023). Assessing the efficacy and safety of hemangioblastoma embolization: a comprehensive systematic review and meta-analysis. J. Clin. Neurosci..

[bib17] Sakamoto N., Ishikawa E., Nakai Y. (2012). Preoperative endovascular embolization for hemangioblastoma in the posterior fossa. Neurol. Med.-Chir..

[bib18] Samartzis D., Gillis C.C., Shih P. (2016). Intramedullary spinal cord tumors: part II-Management options and outcomes. Glob. Spine J..

[bib19] Scarcia L., Kalamarides M., Shotar E. (2024). Direct puncture embolization of a medulla oblongata hemangioblastoma. J. Neuroradiol..

[bib20] Sultan A., Hassan T., Aboul-Enein H. (2016). The value of preoperative embolization in large and giant solid cerebellar hemangioblastomas. Intervent Neuroradiol..

[bib21] Takami H., Graffeo C.S., Perry A. (2022). Presentation, imaging, patterns of care, growth, and outcome in sporadic and von Hippel-Lindau-associated central nervous system hemangioblastomas. J. Neuro Oncol..

[bib22] Takeuchi S., Tanaka R., Fujii Y. (2001). Surgical treatment of hemangioblastomas with presurgical endovascular embolization. Neurol. Med.-Chir..

[bib23] Wan J.-Q., Cui H., Wang Y. (2011). Surgical management of large solid hemangioblastomas of the posterior fossa. J. Clin. Neurosci..

[bib24] Winkler E.A., Birk H., Burkhardt J.-K. (2018). Reductions in brain pericytes are associated with arteriovenous malformation vascular instability. J. Neurosurg..

[bib25] Winkler E.A., Pacult M.A., Catapano J.S. (2022). Emerging pathogenic mechanisms in human brain arteriovenous malformations: a contemporary review in the multiomics era. Neurosurg. Focus.

[bib26] Wu P., Liang C., Wang Y. (2013). Microneurosurgery in combination with endovascular embolisation in the treatment of solid haemangioblastoma in the dorsal medulla oblongata. Clin. Neurol. Neurosurg..

[bib27] Yuh W.T., Han J., Lee C.-H. (2023). The optimal time between embolization and surgery for hypervascular spinal metastatic tumors : a systematic review and meta-analysis. J. Korean Neurosurg. Soc..

