# Supplementary material for: Feasibility and risks of preoperative embolization for CNS hemangioblastoma surgery at a national VHL referral center
Source: Brain Spine. 2026 Oct 12;6:107475. doi: 10.1016/j.bas.2026.107475 (PMC13721012; doi:10.1016/j.bas.2026.107475)
Supplement: Supplementary file 1 [file mmc1.docx]

| **Supplementary Table S1** Surgical outcomes by embolization status and tumor location | | | |
| --- | --- | --- | --- |
| (a) By feasibility | Embolization  n = 21 | Non-feasible  n = 6 | P value |
| Operation time, min, median (IQR) | 271 (230–332) | 372 (345–439) | 0.031 |
| EBL, mL, median (IQR) | 425 (263–1125) | 600 (340–1100) | 0.79 |
| Complete tumor resection, n/N (%) | 17/20 (85.0) | 6/6 (100) | 1.00 |
| (b) By location | Spinal  n = 16 | Intracranial  n = 11 | P value |
| Operation time, min, median (IQR) | 264 (220–342) | 337 (299–484) | 0.034 |
| EBL, mL (IQR) | 350 (250–450) | 620 (320–1200) | 0.25 |
| Values are medians (IQRs), except complete resection, reported as n/N (%). EBL was available for 18/21 embolized and 6/6 non-feasible cases, or 13/16 spinal and 11/11 intracranial cases. Extent-of-resection assessment was unavailable for one embolized case. Statistical comparisons are exploratory.  Abbreviations: EBL, estimated blood loss; IQR, interquartile range. | | | |
