# Supplementary material for: Feasibility and risks of preoperative embolization for CNS hemangioblastoma surgery at a national VHL referral center
Source: Brain Spine. 2026 Oct 12;6:107475. doi: 10.1016/j.bas.2026.107475 (PMC13721012; doi:10.1016/j.bas.2026.107475)
Supplement: Supplementary file 2 [file mmc2.docx]

**Supplementary Table S2. Individual case characteristics and outcomes**

| **Case** | **Year** | **Tumor location** | **Embolic agent** | **Devascular- ization** | **mRS Pre/Post** | **Angiography-related event** |
| --- | --- | --- | --- | --- | --- | --- |
| 1 | 2020 | Obex, intramedullary | Not administered | Non-feasible | 1 → 1 | None recorded |
| 2 | 2019 | C5, intramedullary | Not administered | Non-feasible | 2 → 3 | None recorded |
| 3 | 2021 | Cerebellum | Not administered | Non-feasible | 4 → 4 | None recorded |
| 4 | 2024 | C5–C7, intramedullary | Not administered | Non-feasible | 2 → 3 | None recorded |
| 5 | 2024 | T8, intramedullary | Not administered | Non-feasible | 1 → 1 | None recorded |
| 6 | 2024 | Obex, extramedullary | Not administered | Non-feasible | 3 → 6 Death | None recorded |
| 7 | 2016 | C6/C7, extramedullary | Contour particles (250–355 µm) | Near-complete >75% | 1 → 1 | Femoral artery dissection; no sequelae |
| 8 | 2018 | Right lateral cerebellum | Contour particles (45–150 µm) | Partial 50–75% | 2 → 3 | Labyrinthine artery injury; permanent deficit |
| 9 | 2022 | L3, intra-/extradural extramedullary | Contour particles (250–355 µm) | Low <50% | 1 → 1 | Lumbar arterial branch perforation; no sequelae |
| 10 | 2023 | Medulla oblongata to obex | Contour particles (45–150 µm) | Partial 50–75% | 3 → 5 | Tumor hemorrhage; permanent deficit |
| 11 | 2017 | Left cerebellopontine angle | Contour particles (250–355 µm) | Partial 50–75% | 0 → 0 | None recorded |
| 12 | 2017 | C7/T1, intraforaminal / extradural | Contour particles (150–250 µm) | Partial 50–75% | 1 → 0 | None recorded |
| 13 | 2017 | Right cerebellum | Onyx | Near-complete >75% | 1 → 1 | None recorded |
| 14 | 2018 | Right cerebellum | Onyx | Low <50% | 2 → 1 | None recorded |
| 15 | 2018 | C7/T1, extramedullary | Contour particles (150–250 µm) | Near-complete >75% | 3 → 3 | None recorded |
| 16 | 2018 | Right lateral cerebellum | Contour particles (250–355 µm) | Partial 50–75% | 4 → 3 | None recorded |
| 17 | 2018 | C4, intramedullary | Contour particles (150–250 µm) | Near-complete >75% | 1 → 0 | None recorded |
| 18 | 2013 | Left cerebellopontine angle | PVA particles | Near-complete >75% | 1 → 0 | None recorded |
| 19 | 2005 | T11/T12, extramedullary | PVA particles | Near-complete >75% | 1 → 0 | None recorded |
| 20 | 2012 | T5/T6, extramedullary / foraminal | Contour particles (150–250 µm) | Near-complete >75% | 1 → 0 | None recorded |
| 21 | 2020 | T10/T11, intraforaminal / extradural | PVA particles | Near-complete >75% | 1 → 0 | None recorded |
| 22 | 2008 | C6/C7, intra-/extraforaminal | Contour particles (150–250 µm) | Partial 50–75% | 1 → 0 | None recorded |
| 23 | 2021 | T12/L1, intra-/extraforaminal | Contour particles (150–250 µm) | Near-complete >75% | 1 → 1 | None recorded |
| 24 | 2021 | C7–T2, extramedullary | Contour particles (150–250 µm) | Near-complete >75% | 4 → 3 | None recorded |
| 25 | 2023 | T8, intramedullary | PVA Contour particles | Near-complete >75% | 4 → 4 | None recorded |
| 26 | 2024 | C1/C2, intramedullary | Contour particles (250–355 µm) | Near-complete >75% | 2 → 2 | None recorded |
| 27 | 2023 | Right cerebellum | Contour particles (45–150 µm) | Near-complete >75% | 3 → 3 | None recorded |
| Cases 1–10 corresponds to Table 2. Cases 11–27ND refers to embolic agent not documented. No agent was administered in non-feasible cases. No angiography-related event recorded does not exclude a surgical complication as in case 6, who died after postoperative hemorrhage; this was not counted as an angiography-related complication.  Abbreviations: PVA, polyvinyl alcohol. | | | | | | |
