# Supplementary material for: Feasibility and risks of preoperative embolization for CNS hemangioblastoma surgery at a national VHL referral center
Source: Brain Spine. 2026 Oct 12;6:107475. doi: 10.1016/j.bas.2026.107475 (PMC13721012; doi:10.1016/j.bas.2026.107475)
Supplement: Supplementary file 3 [file mmc3.docx]

**Supplementary Fig. S1** Qualitative operative-field assessment by devascularization category

Proportions of favorable and unfavorable operative-field assessments within each devascularization category. Categories were none (embolization not performed), low (<50%), partial (50–75%), and near-complete (>75%). The exploratory comparison across all four categories yielded P = 1.00.
